# NLRP3 inflammasome pathway involved in the pathogenesis of metabolic associated fatty liver disease

**DOI:** 10.1038/s41598-024-69764-y

**Published:** 2024-08-23

**Authors:** Heba Ahmed Osman, Sawsan M. A. Abuhamdah, Mohammed H. Hassan, Abdelkader Ahmed Hashim, Abdelazeem E. Ahmed, Sameh Salaheldin Elsayed, Samer A. El-Sawy, Mostafa A. Gaber, Marwa Abdelhady

**Affiliations:** 1 Department of Tropical Medicine and Gastroenterology, Faculty of Medicine, South Valley University, Qena, 83523 Egypt; 2 Department of Biopharmaceutics and Clinical Pharmacy, School of Pharmacy, The University of Jordan, P.O. Box: 13380, Amman, 11942 Jordan; 3 Department of Pharmaceutical Sciences, College of Pharmacy, Al Ain University, P.O. Box: 112612, Abu Dhabi, UAE; 4 Department of Medical Biochemistry, Faculty of Medicine, South Valley University, Qena, 83523 Egypt; 5 Department of Biochemistry, Clinical Pharmacy Program, South Valley National University, Qena, 83523 Egypt; 6 Department of Internal Medicine, Faculty of Medicine, South Valley University, Qena, Egypt; 7 Department of Clinical Pathology, Faculty of Medicine, Al-Azhar University, Assiut Branch, Assiut, 71524 Egypt; 8 Department of Medical Biochemistry, Faculty of Medicine, Al-Azhar University, Assiut Branch, Assiut, 71524 Egypt; 9 Department of Restorative Dentistry and Basic Medical Sciences, Faculty of Dentistry, University of Petra, Amman, 11196 Jordan; 10 Department of Medical Biochemistry, Faculty of Medicine, Sohag University, Sohag, Egypt; 11 Department of Diagnostic Radiology, Faculty of Medicine, Al-Azhar University, Assiut Branch, Assiut, 71524 Egypt; 12 Department of Internal Medicine, Faculty of Medicine, Luxor University, Luxor, Egypt

**Keywords:** Inflammasome pathway, IL-1β, NLRP3, Metabolic associated fatty liver disease, RT-qPCR, Molecular biology, Gastroenterology

## Abstract

The prevalence of Metabolic-associated fatty liver disease (MAFLD) has been steadily increasing worldwide, paralleling the global epidemic of obesity and diabetes. It is estimated that approximately one-quarter of the global population is affected by MAFLD. Despite its high prevalence, MAFLD often goes undiagnosed due to the lack of specific symptoms in its early stages. However, as the disease progresses, it can lead to more severe liver-related complications such as fibrosis, cirrhosis, and hepatocellular carcinoma. Therefore, we aimed to investigate the expression levels of the nucleotide-binding oligomerization domain, leucine-rich repeat (LRR)—containing proteins (NLR) family pyrin domain-containing protein 3 [NLRP3] inflammasome pathway components, NLRP3 and interleukin 1β (IL-1β) genes in patients with MAFLD with various degrees of steatosis and fibrosis. Participants were classified into two equal groups; MAFLD group: consisted of 120 patients with different degrees of hepatic fibrosis and steatosis based on fibro scan results. The non-MAFLD group was comprised of 107 participants. Molecular analysis of pyrin domain-containing protein 3 and IL-1β relative gene expressions was performed in the blood of all participants, using Real-time quantitative polymerase chain reaction (RT-qPCR). Patients with post-MAFLD hepatic fibrosis had significantly higher relative gene expression levels of IL-1β and NLRP3; with IL-1β > 1.1 had AUC of 0.919, sensitivity of 88.33, specificity of 96.26, PPV of 96.4, and NPV of 88 and 92.3 accuracy (*p* value < 0.001). NLRP3 > 1.33 had a sensitivity of 97.5, specificity of 99.07, PPV of 99.2, NPV of 97.2, and 98.3 accuracy with an AUC of 0.991 (*p* value < 0.001) as predictors of post-MAFLD hepatic fibrosis.. A significant increase in the mean relative gene expression levels of both IL-1β and NLRP3 found in patients with early fibrosis (F0-F1-2); 31.97 ± 11.8 and 6.76 ± 2.18, respectively; compared with patients with advanced hepatic fibrosis stages (F2-F3); 2.62 ± 3.71 and 4.27 ± 2.99 (*p* < 0.001 each). The present study provides novel evidence for the possible involvement of IL-1β and NLRP3 inflammasome in metabolic-associated fatty liver disease pathogenesis and could be valid markers for the early detection of post-MAFLD hepatic fibrosis.

## Introduction

Metabolic-associated fatty liver disease (MAFLD), which is typically thought of as the hepatic component of metabolic syndrome, can be defined as an abnormal buildup of fat in the liver in the absence of other potential secondary causes of fatty liver. It is currently the most prevalent chronic liver disease worldwide^1–3^.

MAFLD affects approximately one-quarter of the population and is becoming more prevalent, whereas obesity and metabolic syndrome are becoming more common, indicating possible associated inflammation in the liver with subsequent steatosis, fibrosis, and cirrhosis and hepatocellular carcinoma in some cases. The most effective approach to treating MAFLD is to change one's way of life^4,5^.

Inflammasomes are multimeric protein complexes that bind nucleotides and contain leucine-rich repeats (NLR). The most investigated member is NLR family pyrin domain-containing protein 3 (NLRP3) and its activation catalyzes the production of active pro-inflammatory cytokines including interleukin (IL)-1β and IL-18^6,7^.

Inflammasomes play a role in the development and progression of a variety of metabolic diseases, including obesity, diabetes mellitus type II, and atherosclerosis^8^. In addition, NLRP3 has been strongly linked to the development of NASH in the past few years. It has been established that NLRP3 is crucial for the detection of inflammatory stimuli and their conversion to an inflammatory response in the gut-liver axis^9,10^.

The NLRP3 inflammasome has been implicated in steatosis, inflammation, and fibrotic processes in the liver according to a significant amount of evidence from experimental models^11^.

The host defense mechanisms against infection and injury depend on the powerful pro-inflammatory cytokine interleukin-1 (IL-1β)^12^. It is generated and secreted by several innate immune system cell types, including monocytes and macrophages. It is produced in a pro-IL-1β manner. Caspase-1, a pro-inflammatory protease, cleaves pro-IL-1β. The NLRP3 inflammasome is involved in the activation of caspase-1^13^.

The pro-inflammatory cytokine IL-1β plays a critical role in the onset and progression of the non-alcoholic fatty liver disease; from simple steatosis to non-alcoholic steatohepatitis and hepatic fibrosis^14^. In MAFLD and other metabolic disorders, IL-1β is activated via the classical NLRP3 inflammasome activation pathway^15^.

By promoting inflammation and fibrosis and indirectly promoting adipose tissue inflammation and insulin resistance, the NLRP3 inflammasome and caspase-1 are involved in MAFLD pathogenesis^16,17^.

This study aimed to investigate the expression levels of NLRP3 inflammasome and IL-1β genes in the blood of patients with MAFLD with various degrees of fibrosis and steatosis to provide new insights into the possible role of the NLRP3 inflammasome pathway in the pathogenesis of MAFLD.

## Materials and methods

### Study design and participants

Two hundred participants were recruited in this randomized case–control study from outpatients' clinics of Tropical Medicine and Gastroenterology and Internal Medicine Departments, Qena Faculty of Medicine, South Valley University and Internal Medicine Department, Al-Azhar Faculty of Medicine, Al-Azhar University, Assiut branch. Between January 1^st^, 2023, and October 1^st^, 2023. Participants were categorized into two equal groups. The MAFLD group consisted of hundred and twenty patients with different degrees of hepatic fibrosis and steatosis based on the fibro-scan results. The non-MAFLD group was consisted of one hundred and seven non-MAFLD participants.

### Ethical approval

This study was conducted in compliance with the principles of the Declaration of Helsinki. This study was approved by the Institutional Review Board of the Faculty of Medicine, South Valley University, Qena, Egypt (ethical approval code SVU-MED-GIT023-4-23-8-711**)**. All participants provided written informed consents to participate in this study.

### Data collection

The participants were subjected to complete history taking (especially DM, hypertension, abnormal lipid profile, hypothyroidism, obstructive sleep apnea, and polycystic ovarian syndrome), full clinical examination, and calculation of body mass index**.**

Patients with any other liver disease except MAFLD, patients on corticosteroid therapy or any drugs that induce liver disease, alcohol consumption ≥ 30 g/day for men or ≥ 20 g/day for women, all were excluded from the study.

Liver biopsy was not used for the diagnosis of MAFLD in our participants because it carries a high risk to the patients. MAFLD diagnosis depends on abdominal ultrasound, controlled attenuation parameter fibro-scan (with hepatologists with more than five years of experience), and different scoring systems for fibrosis and steatosis assessments. The European Association for the Study of the Liver guidelines (EASL) considered a non-invasive technique suitable for MAFLD diagnosis^18^.

### Investigatory workup

1. A CAP-Fibro scan was used to assess the degree of fibrosis and steatosis (Echosens Fibro Scan 502 touch, France): stages of liver steatosis; S1: (238–260 dm/m); S2: (260–290 dm/m); S3: (290–400 dm/m). The stages of liver fibrosis are stage F0-F1 (7 kPa), F2 (7–8.99 kPa), F3 (9–12.49 kPa), and F4 (12.5 kPa).
2. Fibrosis and steatosis assessments using the following scoring systems.
  Body mass index (BMI) = Weight (kg)/height (m)^2^^19^.
  NAFLD fibrosis score (NFS): Based on 6 parameters (age, BMI, hyperglycemia, platelet count, albumin level, and aspartate transaminase (AST)/alanine transaminase (ALT) ratio)^20^.
  Hepatic steatosis index (HSI) = 8 × (ALT/AST) + BMI + (2, if diabetes mellitus) + (2, if female), with values < 30 and > 36 ruling out steatosis^21^.
  The fatty liver index (FLI) = 0.953 × loge (TG × 88.5) + (0.139 × BMI) + [0.718 × loge (Gamma- glutamyl transpeptidase, GGT)] + (0.053 × WC) − 15.745, with values < 30 excluded and values ≥ 60 confirmed fatty liver disease^22^.
3. Laboratory assays
  Blood samples were obtained from all included patients and controls for assays of complete blood count (CBC), blood urea, serum creatinine, alanine transaminase (ALT), aspartate transaminase (AST), albumin, alkaline phosphatase, bilirubin (total and direct), prothrombin time, prothrombin concentration, INR, hepatitis B surface antigen, and HCV antibody.
4. Molecular analysis of NLRP3 inflammasome and IL-1β gene expressions
  Samples: Two milliliters of antecubital venous blood were withdrawn from all included participants, and each sample was placed in a collecting tube containing the anticoagulant ethylenediaminetetraacetic acid (EDTA) and stored at − 80 °C for later molecular analysis.
  RNA Extraction and Reverse Transcription: Following homogenization with Invitrogen TM TRIzol TM Reagent (Life Technologies Corporation, USA; Catalogue No. 15596026), whole blood samples were treated for total RNA isolation in accordance with the manufacturer's protocol. In this case 0.25 mL from each blood sample was homogenized with 0.75 mL of TRIzolTM Reagent, and then chloroform was added after homogenization. The homogenate was then centrifuged, resulting in separation of the transparent top aqueous layer containing RNA. Isopropanol was used to precipitate the RNA from the aqueous layer. The precipitated RNA was resuspended in RNase-free water and was used for reverse transcriptase-polymerase chain reaction (RT-PCR) after cleaning the contaminants with ethanol. Samples of extracted RNA were stored at − 80 °C following the use of a Nanodrop® (Epoch Microplate Spectrophotometer, Biotek, VA, USA) to determine the concentrations of total RNA. Reverse transcription (RT) of total RNA in extracted RNA samples was performed in a 20 µL reaction using the Thermo Fisher Scientific Applied BiosystemsTM High-Capacity cDNA Reverse Transcription Kit (Catalogue No. 4374966). Prior to qRT-PCR, complementary DNA samples were stored at − 20 °C.
  Real-time quantitative PCR was performed using Thermo Scientific Maxima SYBR Green qPCR Master Mix provided by Thermo Fisher Scientific Baltic (Catalogue no. K0251), which was used along with specific primers to quantify the relative gene expressions of NLRP3 and IL-1β. PCR was carried out in a 20 µL reaction mixture containing 10 µL of Maxima SYBR Green qPCR Master Mix (2X), one µL of amplification primer (from each forward and reverse), 200 ng of template DNA per reaction, and 2 µL of nuclease-free water. The internal control, glyceraldehyde-3-phosphate dehydrogenase (GAPDH), was added to normalize mRNA expression. An Applied Biosystems 7500 Fast Real-Time PCR machine was used for the Real-Time Quantitative polymerase chain reaction (RT-qPCR).
  Using the 2^−∆∆CT^ equation, the threshold cycle value was used to determine relative mRNA expression in each sample. The following primers' 5′–3′ primer sequences were employed: NLRP3 reverse: TGGCTGTTCACCAATCCATGA and forward: GAGGAAAAGGAAGGCCGACA. GADPH is a housekeeping gene that reads as CGTGGAAGGACTCATGACCA forward and GGCAGGGATGATGTTCTGGA reverse. IL-1β reverse: AACACGCAGGACAGGTACAG, and forward: GAGCAACAAGTGGTGTTCTCC. Each primer was determined using the Primer-BLAST software^23,24^.
  The initial denaturation was performed for five minutes at 95 °C. Following the initial denaturation, 40 cycles of 95 °C for 45 s, 49 °C for 45 s, and 72 °C for 1 min were used for GAPDH; 40 cycles of 95 °C for 45 s, 60 °C for 45 s, and 72 °C for 1 min were used for NLRP3; and 40 cycles of 95 °C for 45 s, 55 °C for 45 s, and 72 °C for 1 min for IL-1β. Melting curve profile analysis was used to confirm the amplification of each transcript at the end of each reaction (Fig. S1A, B and C).

### Statistical analysis

Data were analyzed using Statistics Package for Social Sciences (SPSS) version 26 (SPSS Inc., Chicago, IL, USA). Normality tests (Kolmogorov–Smirnov and Shapiro–Wilk tests) were performed, and the data were not normally distributed. Categorical data were described as numbers and percentages (N, %), and differences between the two groups were detected using the chi-square test. Continuous data were expressed as minimum and maximum, mean ± standard deviation (SD), or median (first quartile–third quartile), and differences between the two groups were compared using the Mann–Whitney test for non-parametric data. Pearson and Spearman correlation coefficients were used for the correlation analysis. The ROC curve was used to assess the best cutoff value, sensitivity, specificity, PPV, NPV, and accuracy. Statistical significance was set at *p* < 0.05.

## Results

### Demographic data of studied groups

The current study included 120 adults with MAFLD and 107 non-MAFLD participants, matched for age and sex. In terms of comorbidities, 32.5% of the included participants with MAFLD have diabetes mellitus and or hypertension versus 17.7% among non-MAFLD participants. 69.17% of MAFLD participants were overweight versus 24.3% among non-MAFLD participants. The mean body mass index (BMI) of the MAFLD group (35.56 ± 6.34) was significantly higher than that of the non-MAFLD group (21.23 ± 2.74), (*p* < 0.001). The mean waist circumference in the MAFLD group was significantly higher than that in the non-MAFLD group (102.65 ± 18.34, and 93.5 ± 19.4 respectively, (*p* < 0.001). The mean fibrosis and steatosis scores for MAFLD patients (6.9 ± 2.13, and 294.67 ± 44.0 respectively) were significantly higher than in non-MAFLD group (5.38 ± 1.29, 191.42 ± 15.12 respectively), *p* < 0.001 for both. Detailed anthropometric parameters, fibrosis grades, and steatosis grades were shown in (Table 1).

**Table 1:** Demographic data and fibroscan findings of studied groups.

Clinical and imaging characteristics	MAFLD group (N = 120)	Non-MAFLD group	*p* value
		(N = 107)	
Age (years) (Mean ± SD)	40.93 ± 13.94	39.53 ± 13.31	0.49
Sex (N%)			
Male	25 (20.83%)	32 (29.91%)	0.116
Female	95 (79.17%)	75 (70.09%)	
Hypertension (N%)	26 (21.67%)	12 (11.21%)	0.035*
Diabetes Mellitus (N%)	20 (16.67%)	10 (9.35%)	0.104
BMI (kg/m^2^) (Mean ± SD)	35.56 ± 6.34	21.23 ± 2.74	< 0.001*
Waist circumference (cm) (Mean ± SD)	102.65 ± 18.34	93.5 ± 19.4	< 0.001*
Mid arm circumference (cm) (Mean ± SD)	31.65 ± 5.58	31.36 ± 6.82	0.114
Obesity class			
Normal weight (18.5–24.9 kg/m^2^) (N%)	37 (30.83%)	81 (75.7%)	< 0.001*
Overweight (25 ≥ 30 kg/m^2^) (N%)	83 (69.17%)	26 (24.3%)	
Fibroscan			
Grade of fibrosis by LSM (N%)			
F0	15 (12.5%)	50 (46.73%)	
F0-1	9 (7.5%)	23 (21.5%)	< 0.001*
F1	43 (35.83%)	37 (34.58%)	
F1-2	10 (8.33%)	0 (0%)	
F2	24 (20%)	0 (0%)	
F2-3	7 (5.83%)	0 (0%)	
F3	12 (10%)	0 (0%)	
Score of fibrosis (kpa) (Mean ± SD)	6.9 ± 2.13	5.38 ± 1.29	< 0.001*
Grade of steatosis by CAP (N%)			
S0	0 (0%)	100 (100%)	
S1	20 (16.67%)	0 (0%)	< 0.001*
S1-2	11 (9.17%)	0 (0%)	
S2	31 (25.83%)	0 (0%)	
S2-3	13 (10.83%)	0 (0%)	
S3	45 (37.5%)	0 (0%)	
Score of steatosis (dB/m) (Mean ± SD)	294.67 ± 44.02	191.42 ± 15.12	< 0.001*

*Level of significance < 0.05.

*CAP* controlled attenuation parameter, *LSM* liver stiffness measurement.

### Laboratory data of studied groups

At presentation, patients in the MAFLD group had significantly higher mean hemoglobin, creatinine, ALT, ALP, GGT, and INR levels compared to the non-MAFLD participants, (*p* < 0.001 for all). Also, MAFLD participants have significantly lower mean serum albumin level in comparison with the non-MAFLD group, (*p* = 0.018). In addition, the mean random blood glucose, triglycerides, and VLDL levels in the MAFLD group were significantly higher than those in the non-MAFLD group, (*p* < 0.001, *p* = 0.002, and *p* < 0.001 respectively), as presented in (Table 2).

**Table 2:** Laboratory data of studied groups.

Variables	MAFLD group (N = 120)	Non-MAFLD group (N = 107)	*p* value
Hgb (g/dl) (Mean ± SD)	12.98 ± 1.83	12.06 ± 2.04	< 0.001*
Platelets (10^3^/mm^3^) (Mean ± SD)	281.58 ± 96.24	282.65 ± 70.48	0.794
WBC (× 10^3^/mm^3^) (Mean ± SD)	7.47 ± 3.01	6.9 ± 2.77	0.168
S. creatinine (mg/dl) (Mean ± SD)	1.09 ± 0.29	0.93 ± 0.15	< 0.001*
ALT (IU/L) (Mean ± SD)	31.86 ± 12.85	23.5 ± 6.51	< 0.001*
AST (IU/L) (Mean ± SD)	26.95 ± 12.88	21.28 ± 7.47	0.118
Total bilirubin (mg/dl) (Mean ± SD)	0.87 ± 1.83	0.49 ± 0.32	0.182
Direct bilirubin (mg/dl) (Mean ± SD)	0.39 ± 0.79	0.26 ± 0.25	0.537
Albumin (g/dl) (Mean ± SD)	3.87 ± 0.82	4.11 ± 0.47	0.018*
ALP (U/L) (Mean ± SD)	81.17 ± 31.52	71.85 ± 28.76	0.013*
GGT (U/L) (Mean ± SD)	125.17 ± 54.15	57.42 ± 63.24	< 0.001*
RBG (mg/dL) (Mean ± SD)	142.6 ± 52.95	109.89 ± 21.32	< 0.001*
INR (Mean ± SD)	1.41 ± 0.23	1.11 ± 0.1	< 0.001*
Triglycerides (mg/dL) (Mean ± SD)	165.41 ± 85.62	131.77 ± 54.81	0.002*
Total cholesterol (mg/dL) (Mean ± SD)	181.43 ± 61.18	170.38 ± 41.27	0.182
HDL (mg/dL) (Mean ± SD)	48.33 ± 23.23	59.59 ± 47.2	0.609
LDL (mg/dL) (Mean ± SD)	90.58 ± 49.59	89.04 ± 40.28	0.983
VLDL (mg/dL) (Mean ± SD)	37.77 ± 19.46	24.5 ± 10.13	< 0.001*

*Level of significance < 0.05.

*ALT* alanine aminotransferase, *AST* aspartate aminotransferase, *GGT* γ glutamyl transferase, *HDL* high density lipoprotein, *LDL* low density lipoprotein, *VLDL* very low-density lipoprotein.

### Comparison between relative gene expressions of IL-1β and NLRP3, NAFLD fibrosis score (NFS), hepatic steatosis index, and fatty liver index in the studied groups

Patients in MAFLD group showed significantly higher relative gene expression levels of both IL-1β and NLRP3 in their blood samples (21.45 ± 17.13, and 5.87 ± 2.76 respectively) in comparison with the non-MAFLD group (0.85 ± 0.22, and 0.91 ± 0.2 respectively), (*p* < 0.001for both). In addition, there were significantly higher NAFLD fibrosis score (NFS), hepatic steatosis index (HSI), and fatty liver index (FLI), among MAFLD group compared with the non-MAFLD one, (*p* < 0.001 for all), as showed in (Table 3).

**Table 3:** Comparison between IL-1β and NLRP3 relative gene expressions, NAFLD fibrosis score, hepatic steatosis index and fatty liver index in patients with MAFLD and non-MAFLD group.

Variables	MAFLD group (N = 120)	Non-MAFLD group (N = 107)	*p* value
IL-1β (Mean ± SD)	21.45 ± 17.13	0.85 ± 0.22	˂ 0.001*
NLRP3 (Mean ± SD)	5.87 ± 2.76	0.91 ± 0.2	˂ 0.001*
NFS (Mean ± SD)	− 0.74 ± 1.73	− 1.49 ± 1.68	˂ 0.001*
HSI (Mean ± SD)	37.95 ± 8	31.07 ± 2.93	˂ 0.001*
FLI (Mean ± SD)	75.8 ± 24.02	38.78 ± 23.14	˂ 0.001*

*Level of significance < 0.05.

*NFS* NAFLD fibrosis score, *HIS* Hepatic steatosis index, *FLI* fatty liver index.

Additionally, there were significantly higher relative gene expression levels of both IL-1β and NLRP3 among MAFLD patients with diabetes mellitus or hypertension (n = 39) [21.69 ± 16.16, and 6.3 ± 3.13 respectively] when compared with non-MAFLD participants who have diabetes mellitus or hypertension (n = 19) [0.88 ± 0.18, and 0.99 ± 0.17 respectively], (*p* < 0.001 for both). Also, there were significantly higher relative gene expression levels of both IL-1β and NLRP3 among MAFLD patients with overweight (n = 83) [21.39 ± 17.19, and 5.88 ± 2.77 respectively] when compared with non-MAFLD participants who overweight (n = 26) [0.79 ± 0.28, and 0.84 ± 0.17 respectively], (*p* < 0.001 for both).

There were significant positive correlations between BMI with relative gene expression levels of both IL-1β (r = 0.195, *p* = 0.044), and NLRP3 (r = 0.636, *p* < 0.001) among MAFLD participants.

### Profile of IL-1β and NLRP3 expression levels among the included patients according to fibrosis grade and steatosis grade

There were significant increase in the mean expression levels of both IL-1β and NLRP3 in patients with early fibrosis (F0-F1-2); 31.97 ± 11.8, and 6.76 ± 2.18 respectively when compared with patients have advanced hepatic fibrosis stages (F2-F3); 2.62 ± 3.71, and 4.27 ± 2.99 respectively, (*p* < 0.001 for each).

On the other hand, a significant decrease in mean expression level of IL-1β found in patient with mild steatosis (S0-S1-2); 15.63 ± 14.99 compared with patients with massive hepatic steatosis (S2-S3); 23.48 ± 17.44 (*p* = 0.010). Although the mean relative gene expression level of NLRP3 in patients with mild hepatic steatosis (5.45 ± 2.77) was lower than those with advanced hepatic steatosis (6.01 ± 2.76), this difference didn’t reached a significant level, (*p* = 0.373) as shown in (Table 4).

**Table 4:** Relative IL-1β and NLRP3 gene expressions among the included patients with MAFLD according to fibrosis grade and steatosis grade (N = 120).

Variables	F0 + F0-1 + F1 + F1-2 (N = 77)	F2 + F2-3 + F3 (N = 43)	*p* value
IL-1β (Mean ± SD)	31.97 ± 11.8	2.62 ± 3.71	< 0.001*
NLRP3 (Mean ± SD)	6.76 ± 2.18	4.27 ± 2.99	< 0.001*

Variables	S0 + S1 + S1-2 (N = 31)	S2 + S2-3 + S3 (N = 89)	*p* value
IL-1β (Mean ± SD)	15.63 ± 14.99	23.48 ± 17.44	0.010*
NLRP3 (Mean ± SD)	5.45 ± 2.77	6.01 ± 2.76	0.373

*Level of significance < 0.05.

### Correlation coefficients according to fibrosis & steatosis scores among the included patients

**Figure 1:**
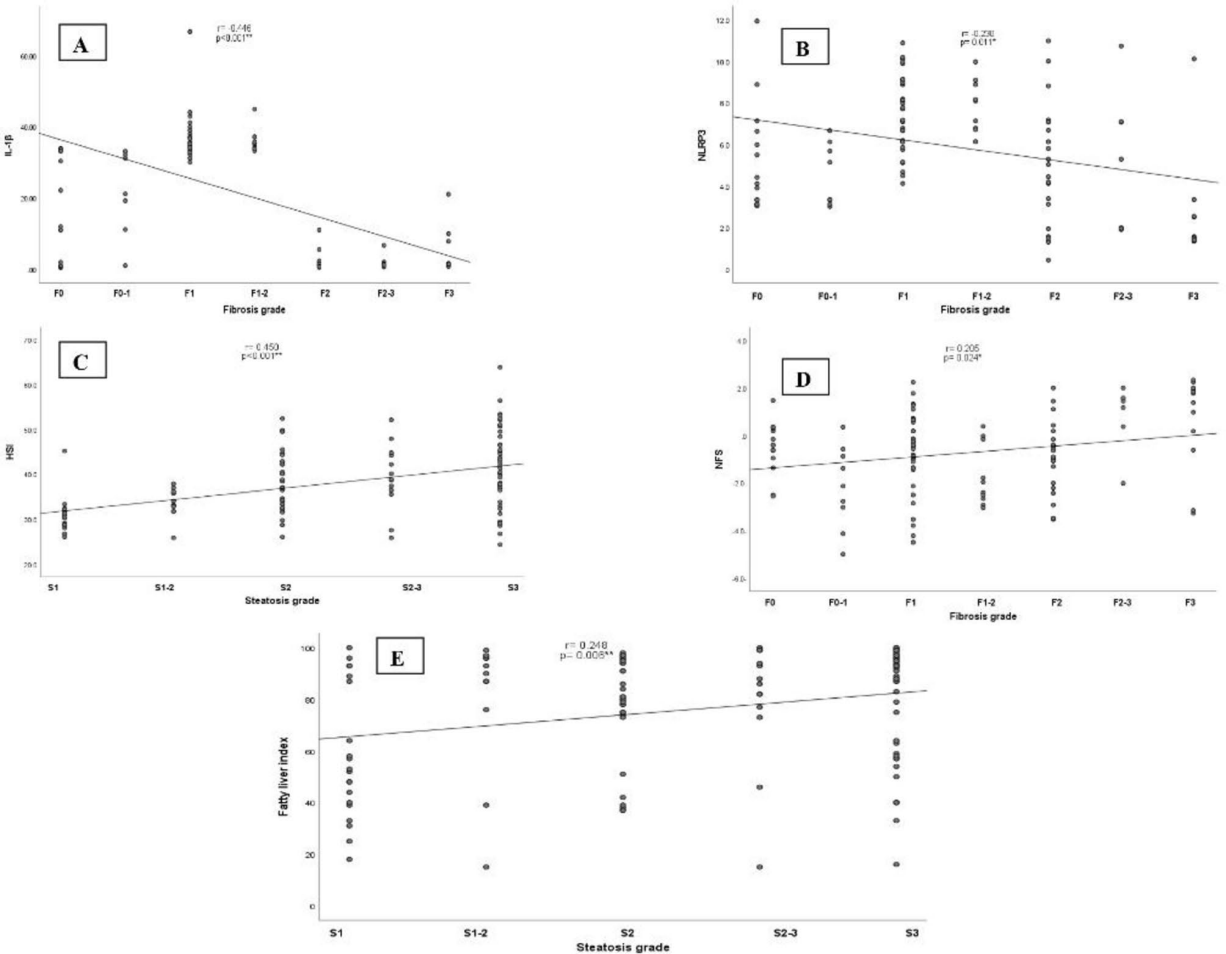
Correlation coefficients according to fibrosis and steatosis grades in MAFLD patients: (**A**) (negative correlation between IL-1β and fibrosis grade), (**B**) (negative correlation between NLRP3 and fibrosis grade), (**C**) (positive correlation between HSI and steatosis), (**D**) (positive correlation between NFS and fibrosis grade), (**E**) (Positive correlation between fatty liver index and steatosis grade).

A significant positive correlation was detected between fibrosis grade and mean MAFLD fibrosis score (NFS) (r = 0.205; *p* = 0.024). In contrast, a significant negative correlations were detected between fibrosis grade and expression levels of each of IL-1β (r = − 0.446, *p* < 0.001), and NLRP3 (r = − 0.230, *p* = 0.011). In addition, significant positive correlations were found between advanced hepatic steatosis grade with each of high hepatic steatosis index (HIS) (r = 0.450, *p* < 0.001), and fatty liver index (r = 0.248, *p* = 0.006), (Fig. 1A–E).

### Linear regression analysis of factors affecting fibrosis score among the included patients

Univariate regression analysis showed that low mean relative gene expression levels of IL-1β (*p* < 0.001) and NLRP3 (*p* = 0.017), and high NFS (*p* = 0.004) were independent factors for hepatic fibrosis in MAFLD patients. While, multivariate logistic regression analysis revealed that low mean relative gene expression level of IL-1β (*p* value < 0.001), and high NFS (*p* = 0.021) significantly associated with hepatic fibrosis in MAFLD patients as presented in (Table 5).

**Table 5:** Linear regression analysis affecting fibrosis score among the included patients.

Variables	Univariate analysis				Multivariate analysis			
	Standardized coefficients beta	*p* value	95.0% confidence interval for B		Standardized coefficients beta	*p* value	95.0% confidence interval for B	
			Lower bound	Upper bound			Lower bound	Upper bound
IL-1β	− 0.441	< 0.001*	− 0.075	− 0.035	− 4.268	< 0.001*	− 0.081	− 0.031
NLRP3	− 0.217	0.017*	− 0.305	− 0.030	0.071	0.487	− 0.101	0.210
NFS	0.264	0.004*	0.108	0.540	0.194	0.021*	0.037	0.439

*Level of significance < 0.05.

*NFS* NAFLD fibrosis score.

### Linear regression analysis affecting of factors affecting steatosis score among the included patients

Regression analysis for hepatic steatosis in patients with MAFLD revealed that BMI (*p* < 0.001), waist circumference (*p* = 0.008), hepatic steatosis index (*p* < 0.001), and fatty liver index (*p* = 0.021) were significantly associated with hepatic steatosis in the univariate analysis, but none of them was detected as an independent factor in the multivariate analysis as revealed in (Table 6).

**Table 6:** Linear regression analysis affecting steatosis score among the included patients.

Variables	Univariate analysis				Multivariate analysis			
	Standardized coefficients beta	*p* value	95.0% confidence interval for B		Standardized coefficients beta	*p* value	95.0% confidence interval for B	
			Lower bound	Upper bound			Lower bound	Upper bound
BMI	0.368	< 0.001*	1.378	3.732	0.201	0.085	− 0.193	2.982
Waist circumference	0.242	0.008*	0.156	1.005	0.154	0.145	− 0.129	0.869
HSI	0.351	< 0.001*	0.992	2.870	0.221	0.091	− 0.199	2.635
FLI	0.211	0.021*	0.060	0.713	− 0.078	0.522	− 0.582	0.297

*Level of significance < 0.05.

*BMI* body mass index, *HIS* hepatic steatosis index, *FLI* fatty liver index.

### Diagnostic accuracy of IL-1β and NLRP3 relative gene expression levels for differentiating between hepatic fibrosis and normal liver

**Figure 2:**
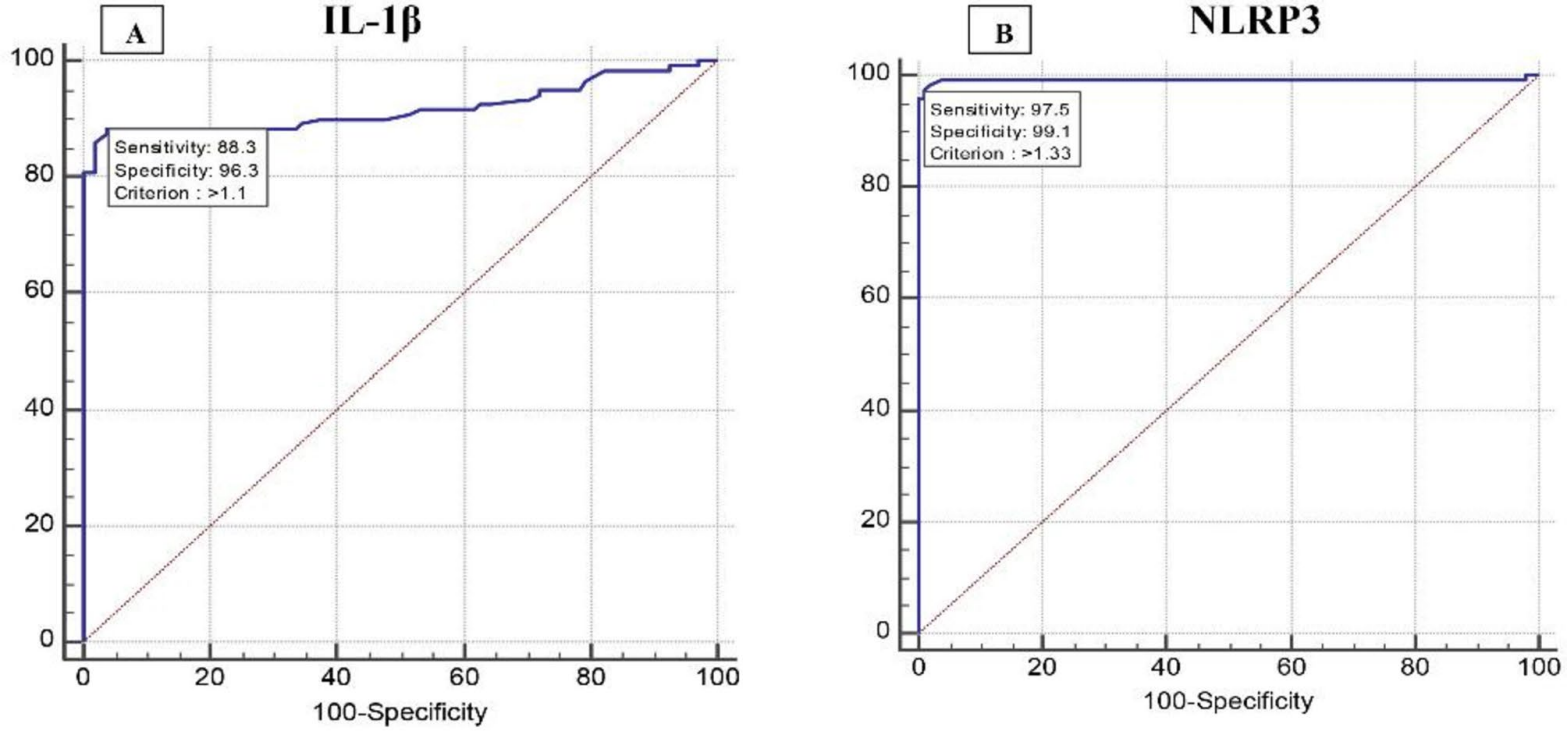
Validity of relative gene expression levels of IL-1β and NLRP3 as predictors of post-MAFLD hepatic fibrosis: (**A**) IL-1β, (**B**) NLRP3. NFS: NAFLD fibrosis score; HIS: Hepatic steatosis index.

The validity of relative gene expression levels of IL-1β and NLRP3 in detecting post-MAFLD hepatic fibrosis revealed that IL-1β at cutoff value > 1.1 had an AUC of 0.919, sensitivity of 88.33%, specificity of 96.26%, positive predictive value (PPV) of 96.4%, negative predictive value (NPV) of 88%, and accuracy of 92.3. NLRP3 at cutoff value > 1.33 had an AUC of 0.991, sensitivity of 97.5%, specificity of 99.07%, PPV of 99.2%, NPV of 97.2%, and accuracy of 98.3 with an (p < 0.001), (Fig. 2A, B).

### Utility of relative gene expression levels of IL-1β and NLRP3 among MAFLD patients to differentiate between early and advanced hepatic fibrosis

**Figure 3:**
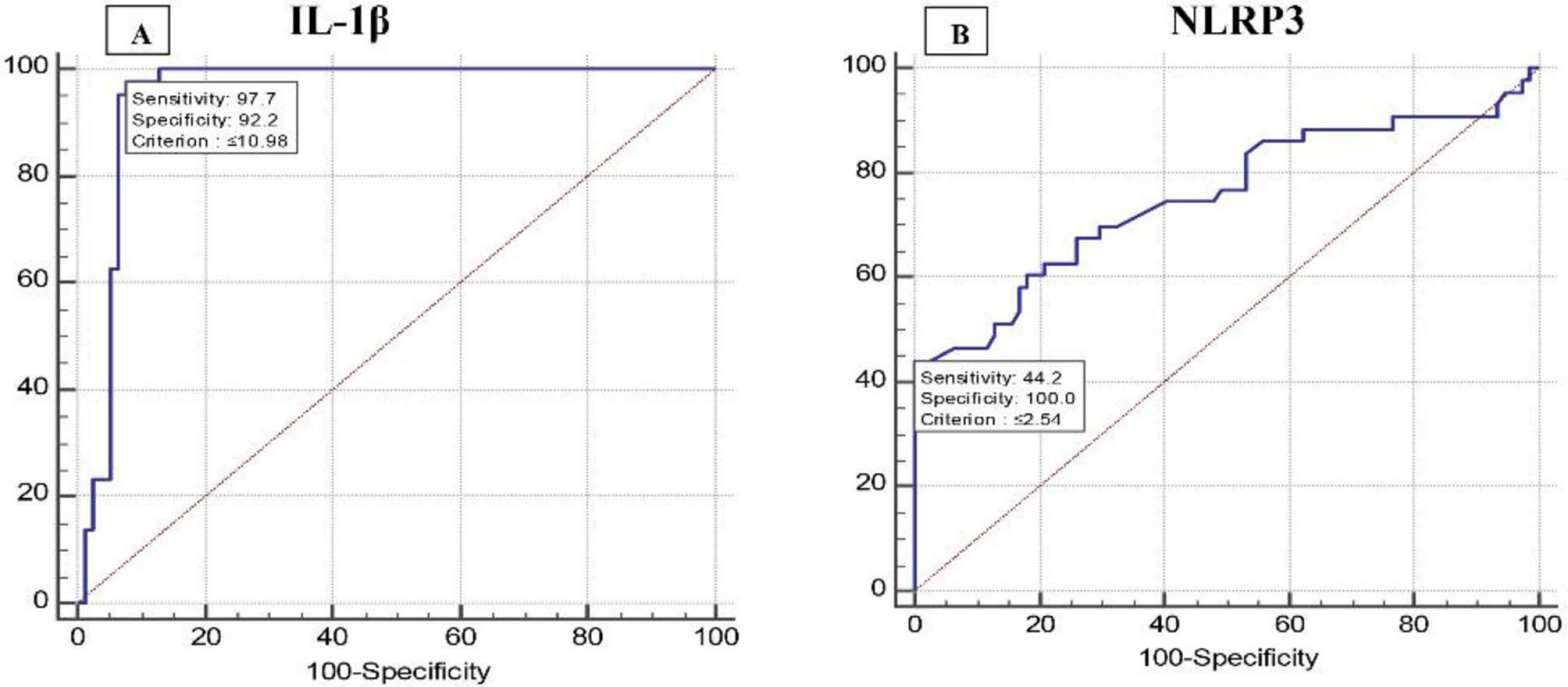
Discriminating utility of IL-1β and NLRP3 relative gene expression levels to differentiate between early and advanced post-MAFLD hepatic fibrosis. (**A**) IL-1β, (**B**) NLRP3.

Receiver operator characteristic (ROC) curve analysis of the relative gene expression levels of IL-1β and NLRP3 to discriminate between early (F0, F0-1, F1, and F1-2) and late (F2 + F2-3 + F3) post-MAFLD hepatic fibrosis revealed that IL-1β at cutoff value ≤ 10.98 had an AUC of 0.949, sensitivity of 97.67%, specificity of 92.21%, PPV of 87.5%, and NPV of 98.6% and accuracy of 94.9. While, NLRP3 at cutoff value ≤ 2.54 had an AUC of 0.754, sensitivity of 44.19%, specificity of 100%, PPV of 100%, NPV of 76.2%, and accuracy of 72.1, (Fig. 3A, B).

### Validity of relative gene expression levels of IL-1β and NLRP3 among MAFLD patients to discriminate between early and advanced hepatic steatosis

**Figure 4:**
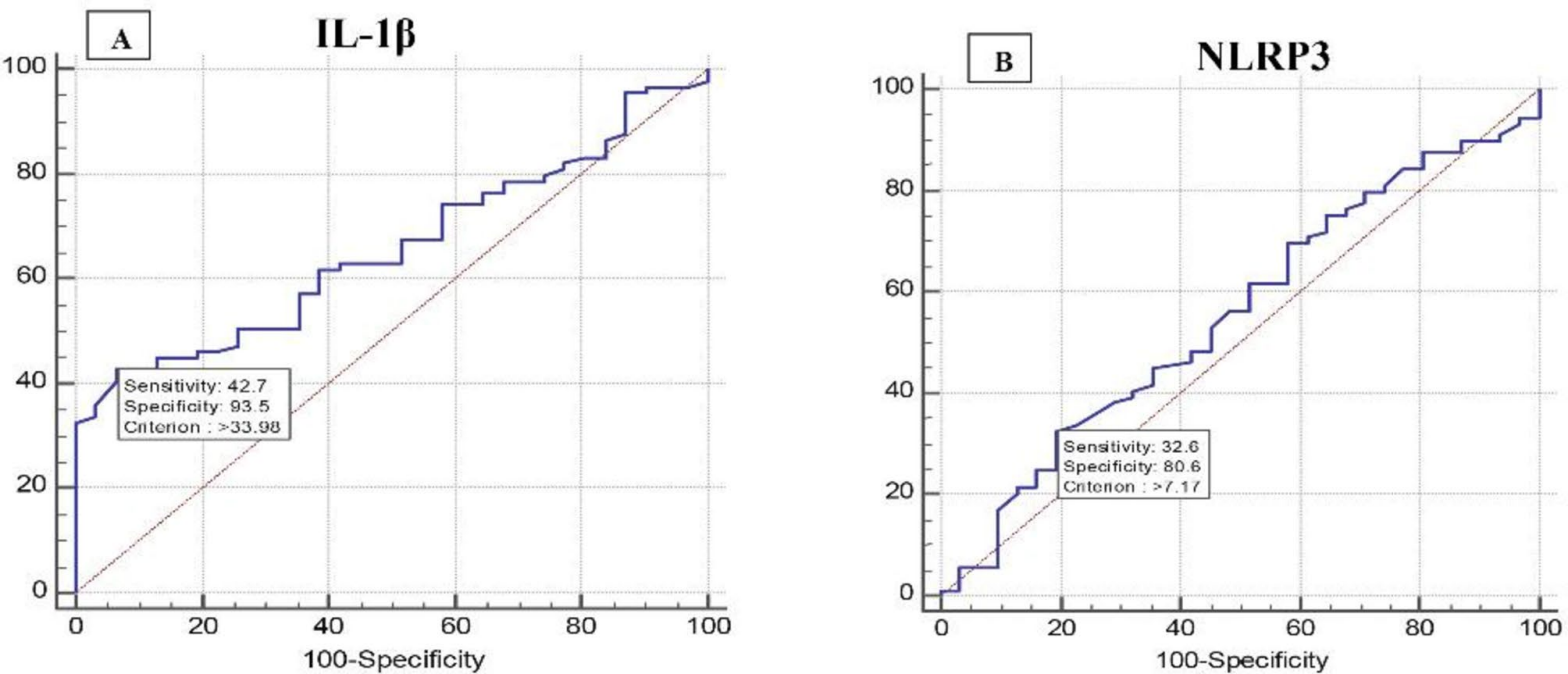
ROC curve of IL-1β and NLRP3 expression levels in discriminating patients with early and patients with advanced post-MAFLD hepatic steatosis. (**A**) IL-1β, (**B**) NLRP3.

ROC curve analysis for relative gene expression levels of both IL-1β and NLRP3 to discriminate between early (S0, S1, S1-2) and late (S2 + S2-3 + S3) post-MAFLD hepatic steatosis showed that IL-1β at cutoff value > 33.98 had an AUC of 0.656, sensitivity of 42.7%, specificity of 93.55%, PPV of 95%, and NPV of 36.2% and accuracy of 68.1. While, NLRP3 at cutoff value > 7.17 had an AUC of 0.554, sensitivity of 32.58%, specificity of 80.65%, PPV of 82.9%, NPV of 29.4%, (Fig. 4A, B).

## Discussion

An increasing cause of chronic liver disease and its implications is MAFLD, affecting approximately 25% of the general population and over 50% of individuals with dysmetabolic conditions^4^.

In this study, we evaluated the possible pathogenic roles of NLRP3 and IL-1β in the development of steatosis and fibrosis in patients with MAFLD. Patients with obesity exhibit a distinctive metabolic profile imbalance that is linked to significant changes in insulin sensitivity, inflammatory responses, and other molecular modifications, increasing the risk of developing metabolic diseases^25^.

In this study, the prevalence of MAFLD was higher in females, and participants diagnosed with MAFLD had a higher prevalence of hypertension, type II diabetes mellitus, BMI, and waist circumference. This aligns with the findings of Fan et al.^26^ in their research study of 5377 patients, they observed that patients with MAFLD had an increased percentage of female patients and a greater number of cases with hypertension, type II diabetes mellitus, high BMI, and larger waist circumference than the non-MAFLD group. In agreement with our findings, Abdulmaseh et al.^27^, reported that 42% of the included patients with cardiovascular disease (CVD) have MAFLD concluding that MAFLD could be a strong independent risk factor for development of CVD .Similar findings have been reported by Sayed et al.^28^. It is a perplexing phenomenon that chronic “sterile” inflammation can emerge during obesity without the presence of an overt infection or autoimmune disease^29^. In the current study, we observed significant positive correlations between BMI with the relative gene expression levels of both NLPR3 and IL-1β in the blood of individual with MAFLD were 69.17% of them have overweight. These findings were consistent with recent studies that found the NLRP3 inflammasome to be active and to be a major contributor to disorders associated with obesity^29–31^.

For the purpose of determining and diagnosing steatosis, FibroScan's controlled attenuation parameter (CAP) value is an acceptable substitute for liver biopsy^32,33^. In this study, in patients with MAFLD, the mean hepatic fibrosis score by LMS was 6.9 ± 2.13 and the mean hepatic steatosis score by CAP was 294.67 ± 44.02 with significant difference from that in non-MAFLD participants. This was accompanied by Yoo et al.^34^ data, which demonstrated a strong correlation between CAP and the degree of hepatic steatosis. For advanced steatosis, a cutoff CAP value of 298.5 dB/m was acceptable. For hepatic fibrosis, it was 5.7 kPa.

At presentation, our included patients with MAFLD showed significantly higher mean hemoglobin levels than non-MAFLD participants. Although WBCs counts were higher in the MAFLD group and platelet counts were lower in the MAFLD group than in the non-MAFLD group, no significant differences were observed. Juárez-Hernández et al.^35^ and Zhang et al.^36^ have demonstrated this phenomenon. Furthermore, the notable increase in hemoglobin levels may indicate that hemoglobin plays a protective role by acting as an antioxidant and mitigating the negative effects of this illness.

Among the MAFLD patients we included, there was a substantial decline in the mean albumin level with significant increase in the mean serum ALT, ALP, GGT, and INR levels, also a significant impairment in renal function was present, as indicated by elevated serum creatinine.

Numerous studies have reported similar findings. Additionally, poor prognosis and disease progression were thought to be associated with low albumin levels^37–40^.

The mean serum triglyceride, VLDL, and mean random blood glucose levels of MAFLD patients were all elevated. Although MAFLD patients had lower HDL levels than non-MAFLD patients, the difference was not statistically significant. This was in line with the findings of Mansour-Ghanaei et al.^41^ who observed abnormalities in the lipid profile and blood sugar levels of patients with MAFLD. In addition, they advocated investigating lipid profiles and biochemical markers of patients diagnosed with MAFLD using ultrasonography.

MAFLD is the most common cause of liver-related events globally, although only a small percentage of patients develop cirrhosis and end-stage liver disease. According to existing data, the degree of liver fibrosis has the strongest predictive influence in MAFLD and is independently linked to hepatic outcomes^42^. Although liver biopsy remains the "gold standard" for determining disease severity, an increasing number of alternatives based on imaging or laboratory testing have been developed^43^.

We analyzed three easy-to-use measures (NFS, FLI, and HSI) that were previously validated in patients with MAFLD. We observed that our MAFLD patients had significantly higher values than non-MAFLD participants. According to Bernstein et al.^44^, the NAFLD fibrosis score (NFS) is a simple assembly scoring method that effectively distinguishes patients with hepatic fibrosis from those without fibrosis in MAFLD. Additionally, a study by Kang et al.^45^ the fatty liver index (FLI) is a valid scoring system that assesses the quantity of fat in the liver, and a high FLI is strongly linked to an increased risk of developing hepatocellular carcinoma^45^. In another study by Lee et al.^21^ who concluded that the HSI is simple and effective NAFLD screening tool with a value above 36 establish MAFLD, while values below 30 rules out MAFLD. It can also be used to assess whether lifestyle changes are necessary or not.

To the best of our knowledge, this is the first study to assess IL-1β and NLPR3 relative gene expression levels by qPCR in the blood of patients with varying degrees of hepatic fibrosis and steatosis associated with MAFLD. According to our findings, patients in the MAFLD group had significantly higher expression levels of both NLPR3 and IL-1β.

This was consistent with a previous study that found IL-1β to be a key mediator of inflamm-aging or age-related systemic, chronic, low-grade inflammation^46^. Both parenchymal and non-parenchymal cells can produce significant levels of IL-1β after liver damage. Through a variety of mechanisms, including steatosis promotion, insulin signaling disruption, fibrotic protein synthesis by stellate cells, and neutrophil recruitment, IL-1β contributes to the pathogenesis of non-alcoholic steatosis^47^. Additionally, as previously established, pro-IL-1β is cleaved into its active form by caspase-1, which is activated by the cytosolic protein complex known as the NLRP3 inflammasome. Kupffer cells, macrophages, other inflammatory cells, and parenchymal cells in the liver are the primary cells that express the NLRP3–caspase-1 complex^14^. The activation of the NLRP3 inflammasome favors the onset of steatohepatitis and liver fibrosis^48^.

In the current study, we compared the expression levels of NLRP3 and IL-1β in MAFLD individuals with varying degrees of hepatic fibrosis and steatosis and found a considerable lowering in their expression levels was linked to advanced fibrosis. Additionally, MAFLD patients with advanced steatosis showed a significant increase in the expression IL-1β levels. Although there was an increase in the expression levels of NLRP3 in advanced steatosis, this increase was not statistically significant.

This was in accordance with the results of Baiocchi et al. ’study, who found that a disproportionate relationship between the development of fibrosis and excessive histological necro-inflammation with the rapid progression toward hepatic fibrosis may be caused by factors other than liver inflammation^49^. In our study, IL-1β and NLRP3 expression levels were negatively correlated with MAFLD fibrosis grade, whereas NFS was positively correlated with MAFLD fibrosis grade. Additionally, the HSI and fatty liver index were positively correlated with hepatic steatosis. Therefore, decreasing expression level of IL-1β and NLRP3 and increasing values of NFS can be used as non-invasive methods to assess advanced hepatic fibrosis. Furthermore, high HSI and fatty liver index measures can be used as non-invasive indices to assess hepatic steatosis.

Pearson's correlation analysis showed that post-MAFLD hepatic fibrosis positively correlated with low expression levels of IL-1β, low expression levels of NLRP3, and high NFS values. Furthermore, low expression IL-1β levels and high NFS values were independent predictors of hepatic fibrosis in MAFLD patients using multiple stepwise linear regression analysis. As reducing IL-1β may be associated with the suppression of inflammation, which may be predictive of hepatic fibrosis, this could account for the potential mechanism connecting IL-1β to hepatic fibrosis in patients with MAFLD. An additional connection between increased NFS and advanced hepatic fibrosis is that the latter is substantially correlated with abnormalities in AST, ALT, platelets, and albumin, which are closely linked to advanced hepatic illness.

In this study, hepatic steatosis in patients with MAFLD was evaluated using linear regression analysis, and was found to be independently correlated with high BMI, increased waist circumference, high HSI, and high FLI. These results align with earlier research that showed a robust positive association between BMI, WC, HSI, and FLI and advanced hepatic steatosis^50,51^. Validation of IL-1β at a level > 1.1 for discrimination between post-NAFLD hepatic fibrosis and non-fibrotic liver in patients with different body mass indices and metabolic risk factors showed a sensitivity of 88.33%, specificity of 96.26%, PPV of 96.4%, NPV of 88%, and accuracy of 92.3%, with a significant p-value. At the same time NLRP3 > 1.33 significantly discriminating between post-NAFLD hepatic fibrosis and non-fibrotic liver in patients with different body mass index and different metabolic risk factors with a sensitivity of 97.5%, specificity of 99.07%, PPV of 99.2%, NPV of 100% and accuracy of 98.3%. This was elucidated by research done by Mirea et al. and Sheriff et al. regarding NLRP3 and IL-1β as inflammasomes involved in the pathogenesis of MAFLD^14,52^.

To the best of our knowledge, this study is the first to use the cutoff point for IL-1β and NLRP3 inflammasomes to discriminate between early and advanced hepatic fibrosis and steatosis in patients with MAFLD. In this study IL-1β and NLRP3 were more sensitive than steatosis in the detection of fibrosis. IL-1βat a level ≤ 10.98 had sensitivity of 88.33%, specificity of 96.26% in differentiating between early and advanced post-MAFLD hepatic fibrosis with significant p.value. However, IL-1β had 42.7% sensitivity and 93.55 specificity in discriminating between early and late post-MAFLD hepatic steatosis, with a significant p-value. Furthermore, NLRP3 ≤ 2.54 was able to significantly distinguish between early and severe post-MAFLD hepatic fibrosis with a sensitivity of 44.19% and a specificity of 100%. NLRP3 > 7.17 demonstrated 32.58 sensitivity and 80.65% specificity in distinguishing between early and advanced hepatic steatosis, but with a non-significant *p* value.

## Conclusion

The findings of the current study provide new evidence supporting the important role of NLRP3 inflammasomes in MAFLD pathogenesis and highlight their potential for early detection of post-MAFLD liver fibrosis. Thus, circulating relative gene expression levels of inflammasome components (NLRP3 and IL-1β) can be used to stratify MAFLD severity. In addition, future research on the effect of inhibitors of this pathway on the management of MAFLD is recommended.

## Limitations of the study

One of the study’s limitations was lack of western blot analysis or ELISA assays of the studied inflammasome components (NLRP3 and IL-1β) and other involved components such as gasdermin D (GSDMD), caspase-1 or IL-18 expressions among the included participants.

### Supplementary Information

Supplementary Information.

## Data Availability

The datasets used and/or analyzed during the current study are available from the corresponding author upon reasonable request, after obtaining the permission of our institute.
