# Supplementary material for: NLRP3 inflammasome pathway involved in the pathogenesis of metabolic associated fatty liver disease
Source: Sci Rep. 2024 Aug 23;14:19648. doi: 10.1038/s41598-024-69764-y (PMC11344024; doi:10.1038/s41598-024-69764-y)
Supplement: Supplementary file 1 — Supplementary Information. [file 41598_2024_69764_MOESM1_ESM.docx]

**Supplementary data**

**NLRP3 inflammasome pathway involved in the pathogenesis of metabolic associated fatty liver disease**

**Heba Ahmed Osman^1^, Sawsan M A Abuhamdah^2,3^,Mohammed H. Hassan^4,5*^, Abdelkader Ahmed Hashim^6^, Abdelazeem E. Ahmed^7^, Sameh Salaheldin Elsayed^8^, Samer A. El-Sawy^9,10^, Mostafa A. Gaber^11^, Marwa Abdelhady^12^**

^1^Department Of Tropical Medicine and Gastroenterology, Faculty of Medicine, South Valley University, Qena 83523, Egypt.

^2^Department of Biopharmaceutics and Clinical Pharmacy, School of Pharmacy, The University of Jordan, Amman, Jordan. P.O.Box: 13380, Amman 11942, Jordan.

^3^Department of Pharmaceutical Sciences, College of Pharmacy, Al Ain University, Abu Dhabi, United Arab Emirates. P.O.Box: 112612 Abu Dhabi, UAE.

^4^Department of Medical Biochemistry, Faculty of Medicine, South Valley University, Qena 83523, Egypt.

^5^Department of Biochemistry, Clinical Pharmacy Program, South Valley National University, Qena 83523, Egypt.

^6^Department of Internal Medicine, Faculty of Medicine, South Valley University, Qena, Egypt.

^7^Department of Clinical Pathology, Faculty of Medicine, Al-Azhar University, Assiut Branch, Assiut 71524, Egypt.

^8^Department of Medical Biochemistry, Faculty of Medicine, Al-Azhar University, Assiut Branch, Assiut 71524, Egypt.

^9^Department of Restorative Dentistry and Basic Medical Sciences, Faculty of Dentistry, University of Petra, Amman 11196, Jordan.

^10^Department of Medical Biochemistry, Faculty of Medicine, Sohag University, Sohag, Egypt

^11^Department of Diagnostic Radiology, Faculty of Medicine, Al-Azhar University, Assiut Branch, Assiut 71524, Egypt.

^12^Department of Internal Medicine, Faculty of Medicine, Luxor University, Luxor, Egypt.

***Corresponding author***: *To whom correspondence should be addressed:

Dr. Mohammed H. Hassan, Department of Medical Biochemistry, Faculty of Medicine, South Valley University, Qena 83523, Egypt . . <https://orcid.org/0000-0003-2698-9438>

E-mail: [Mohammedhosnyhassaan@yahoo.com](mailto:mohammedhosnyhassaan@yahoo.com); [mohammedhosnyhassaan@med.svu.edu.eg](mailto:mohammedhosnyhassaan@med.svu.edu.eg); Tel.: +201098473605


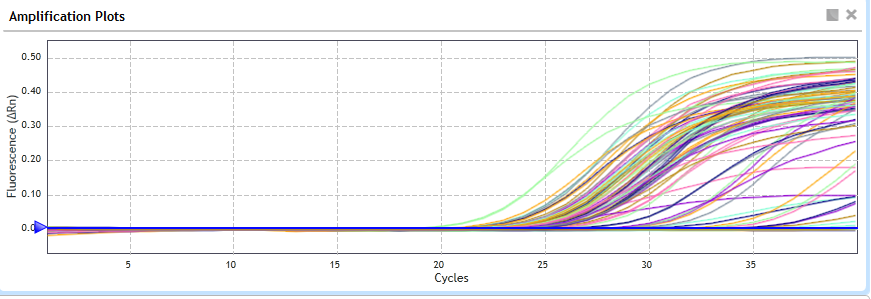

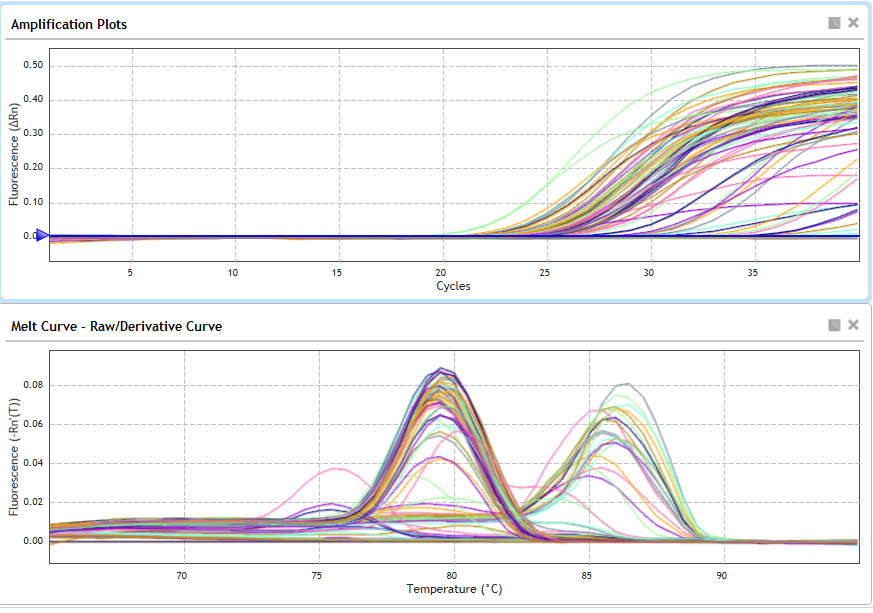


C

A

B

**Fig. S 1.** Melting-curve profile analysis was used to confirm the amplification of each transcript of IL-1β and NLRP3 at the conclusion of each reaction.
